# Engineering of an Anti-Inflammatory Peptide Based on the Disulfide-Rich Linaclotide Scaffold

**DOI:** 10.3390/biomedicines6040097

**Published:** 2018-10-06

**Authors:** Claudia Cobos, Paramjit S. Bansal, Linda Jones, Phurpa Wangchuk, David Wilson, Alex Loukas, Norelle L. Daly

**Affiliations:** Centre for Biodiscovery and Molecular Development of Therapeutics, AITHM, James Cook University, Cairns 4870, Australia; claudia.cobos@my.jcu.edu.au (C.C.); paramjit.bansal@jcu.edu.au (P.S.B.); linda.jones1@jcu.edu.au (L.J.); phurpa.wangchuk@jcu.edu.au (P.W.); david.wilson4@jcu.edu.au (D.W.); alex.loukas@jcu.edu.au (A.L.)

**Keywords:** anti-inflammatory peptides, disulfide-rich peptides, inflammatory bowel disease

## Abstract

Inflammatory bowel diseases are a set of complex and debilitating diseases, for which there is no satisfactory treatment. Peptides as small as three amino acids have been shown to have anti-inflammatory activity in mouse models of colitis, but they are likely to be unstable, limiting their development as drug leads. Here, we have grafted a tripeptide from the annexin A1 protein into linaclotide, a 14-amino-acid peptide with three disulfide bonds, which is currently in clinical use for patients with chronic constipation or irritable bowel syndrome. This engineered disulfide-rich peptide maintained the overall fold of the original synthetic guanylate cyclase C agonist peptide, and reduced inflammation in a mouse model of acute colitis. This is the first study to show that this disulfide-rich peptide can be used as a scaffold to confer a new bioactivity.

## 1. Introduction

Peptides display a range of potentially useful biological functions such as anti-inflammatory [1], anti-cancer [2], anti-HIV [3], antimicrobial [4], and insecticidal [5] activities, among others. Peptides as drug leads have a range of advantages over small molecules and proteins, including target specificity, low toxicity, and immunogenicity, but one major limitation for small, unstructured peptides is a lack of stability in vivo [6]. Small, unconstrained peptides can be degraded within a few minutes in the blood, which decreases their potential as therapeutic agents.

Despite the stability issues that can be associated with peptides, there are several examples of peptides used in the clinic, including cyclosporine, Prialt^®^ (Ziconotide), and linaclotide. Cyclosporine is a 12-residue cyclic peptide that has famously revolutionized organ transplant therapy due to its potent immunosuppressant activities [7]. Prialt is a synthetic version of the cone-snail venom peptide MVIIA and is currently used for the treatment of chronic pain [8]. This cone-snail venom peptide is a calcium channel antagonist, containing 25 residues and three disulfide bonds in a cystine knot motif [9]. Linaclotide, a 14-amino-acid peptide with three disulfide bonds, interacts with guanylate cyclase-C, generating cyclic guanosine monophosphate (cGMP), and is currently in clinical use for patients with chronic constipation or irritable bowel syndrome [10]. Linaclotide is administered orally and improves bowel function and abdominal discomfort [11]. These three peptides are constrained either by a cyclic backbone or disulfide bonds, highlighting the advantages of using covalent constraints to improve the therapeutic potential of peptides.

We have recently used the cyclic peptide SFTI-1 as a scaffold to stabilize a tripeptide with anti-inflammatory activity [12]. SFTI-1 is a 14-residue cyclic peptide isolated from the seeds of sunflowers (*Helianthus annuus*), and is one of the most potent trypsin inhibitors known [13]. It contains two short antiparallel β-strands linked by a single disulfide bond, and the structure contains a network of hydrogen bonds which makes it extremely stable for engineering modifications [14,15]. MC-12 is a tripeptide originally derived from annexin A1, which we grafted into the binding loop of SFTI-1. Our grafted peptide, termed cyc-MC12, exhibited a significantly improved therapeutic efficacy in a murine model of chemically-induced acute colitis, and in vitro stability compared to MC-12 [12].

Colitis is one of the major forms of inflammatory bowel diseases (IBD), which are a set of debilitating chronic inflammatory disorders of the gastrointestinal tract [16]. Current treatments are not satisfactory, and consequently new drug leads are being sought from a range of sources, including small molecules from plants and bioactive regions of larger proteins [17,18,19,20]. To further explore the potential of using disulfide-rich/cyclic peptide scaffolds for IBD applications, we have used the linaclotide scaffold for grafting the MC-12 sequence. Given that the linaclotide scaffold is highly constrained and orally active, we decided to explore its potential as a scaffold for engineering novel bioactivities. Linaclotide regulates guanylate cyclase C (GCC) and is used in the treatment of irritable bowel syndrome (IBS), but has not been demonstrated to regulate autoimmune diseases such as inflammatory bowel disease (IBD), making this study the first to examine its potential as a scaffold in IBD.

## 2. Experimental

### 2.1. Peptide Synthesis and Purification

The peptides were synthesized using fluorenylmethyloxycarbonyl (Fmoc) chemistry-based solid-phase peptide synthesis with 2-chlorotrityl chloride resin on a 0.1 mmole scale. Amino acids (2 equiv.) were activated in 5 equiv. of *N*,*N*,*N*′,*N*′-tetramethyl-*O*-(1H-benzotriazol-1-yl)uranium hexafluorophosphate and 10 equiv. of *N*,*N*-diisopropylethylamine in dimethylformamide (DMF) (1.5 mL). Deprotection was carried out in two repetitions: Starting with 2 min of 20% piperidine in DMF (5 mL), followed by 3 min of the same solution. The C-terminal amino acid was coupled manually to the resin, and the remainder of the peptide was assembled using a Protein Technologies PS3 synthesizer following the Fmoc approach. Peptides were cleaved from the resin using a mixture of trifluoroacetic acid (TFA)/water/triisopropylsilane (95:2.5:2.5) for 2–3 h. Each peptide was precipitated with diethylether after cleavage, dissolved in 50% acetonitrile/0.05% TFA, and then lyophilised. RP-HPLC was used for purification on a C18 preparative column (Phenomenex Jupiter 250 × 21.2 mm, 10 µm, 300 Å) with 1% gradient of solvent B (solvent A: 0.05% TFA; solvent B: 90% acetonitrile, 0.05% TFA). Masses were analysed using matrix-assisted laser desorption ionization time-of-flight (MALDI-TOF) mass spectrometry. The reduced peptides were oxidised in 0.1 M ammonium bicarbonate (pH 8.5) buffer containing 2 mM reduced glutathione for 24 h at room temperature. The oxidized peptides were purified using RP-HPLC and masses analysed using MALDI-TOF mass spectrometry. 

### 2.2. NMR Spectroscopy and Structural Analysis

After purification, the peptides were resuspended at a final concentration of ~0.2 mM in 90% H_2_O:10% D_2_O, or 100 mM deuterated SDS dissolved in 90% H_2_O:10% D_2_O. 2D ^1^H-^1^H TOCSY, ^1^H-^1^H NOESY, ^1^H-^1^H DQF-COSY, ^1^H-^15^N HSQC, and ^1^H-^13^C HSQC spectra were acquired at 290 K using a 600 MHz AVANCE III NMR spectrometer (Bruker, Karlsruhe, Germany). NOESY spectra were acquired with mixing times of 200–300 ms, and TOCSY spectra were acquired with isotropic mixing periods of 80 ms. Standard Bruker pulse sequences were used with an excitation sculpting scheme for solvent suppression. Spectra were referenced to internal 4,4-dimethyl-4-silapentane-1-sulfonic acid (DSS). 

NMR assignments were made using established protocols [21], and the secondary shifts derived by subtracting the random coil αH shift from the experimental αH shifts [22]. The 2D NOESY spectra of MC12-linaclotide were automatically assigned and an ensemble of structures calculated using the program CYANA [23]. Dihedral-angle restraints were derived based on the J_αN_ coupling constants measured from the one-dimensional spectra. The final structures were visualized using MOLMOL [24]. 

### 2.3. TNBS Colitis Assay

The animal experiments were conducted in accordance with the James Cook University Animal Ethics Committee approved guidelines. Five male BALB/c were used for each group (five weeks old). Mice were purchased from the Animal Resources Centre (Perth, Australia) and housed in the animal care facility unit at James Cook University in Cairns under specific pathogen-free conditions, with unlimited access to food and water in their cages. Mice were divided randomly into four groups: Naïve, 2,4,6-trinitrobenzenesulfonic acid (TNBS, Saint Louis, MO, USA), MC12-linaclotide plus TNBS, and linaclotide plus TNBS. Mice received intraperitoneal (i.p) injections of peptides at a dosage of 3 mg/kg body weight. Prior to intra-rectal administration of TNBS, mice were anaesthetized using mild ketamine/xylazine solution. After anaesthesia, each mouse received 100 μL of 5% (*w*/*v*) TNBS solution in 60% ethanol by intra-colonic instillation using a 20 gauge soft catheter (Terumo, Tokyo, Japan), which was inserted into the anus and up to the colon. Mice were monitored daily for body weight, piloerection, survival, decreased motor activity, rectal bleeding, and stool consistency. Mice were humanely euthanised using gas asphyxiation, where CO_2_ was applied directly to the individual cage for approximately 1.5 min, animals were removed from the cage, and death was confirmed. After the cull, the macroscopic pathology score was calculated for each colon. Briefly, colons were harvested, opened longitudinally, and washed with sterile phosphate buffer saline. The tissues were assessed for changes in macroscopic appearance, and scored for pathological changes as follows: adhesion (0 to 3), bowel wall thickening (0 to 3), mucosal oedema (0 to 3), ulceration (0 to 3), and colon length as described previously [25]. All animal experiments were conducted in duplicate to ensure reproducibility of the findings.

### 2.4. Tissue p-IκB-α (Ser32) and p-NF-κB p65 (Ser536) Measurements

The levels of phosphorylated inhibitors phosphorylated nuclear factor kappa-light-chain-enhancer of activated B cells p65 (Ser536) (phospho-NF-κB p65 (Ser536)) and phosphorylated inhibitor of κB-α (Ser32) (phospho-IκBα (Ser32)) were measured using tissue homogenates prepared at 4 °C using phosphate buffer as per the manufacturer’s instructions using a manual tissue grinder. Results were determined by ELISA using PathScan® kits (Cell Signalling Technology, Danvers, IL, USA), and absorbances were read using a POLARstar Omega spectrophotometer (BMG Labtech, Ortenberg, Germany). Statistical analyses were performed using four groups of mice (naïve, TNBS only, linaclotide + TNBS, and MC12-linaclotide + TNBS), with a total of 20 mice. Error bars represent ± SEM.

## 3. Results

### 3.1. Peptide Design and Synthesis

**Figure 1:**
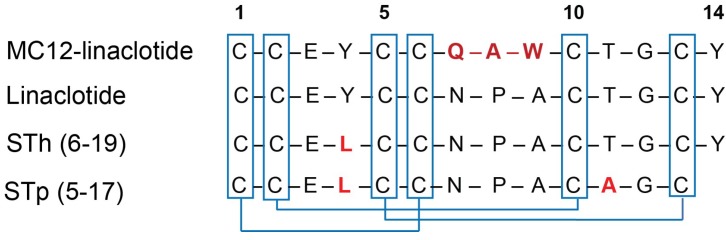
Sequences of MC-12-linaclotide, linaclotide, STh (6-19) and STp (5-17), linaclotide-related peptides. The differences in sequences are highlighted in red. The disulfide bonds are highlighted in blue.

The linaclotide sequence contains three inter-cysteine loops, comprising two or three residues. To avoid changing the inter-cysteine loop sizes, we grafted MC-12 into the second loop, which contains three residues, as shown in Figure 1. Linaclotide and the grafted peptide (MC12-linaclotide) were synthesised by Fmoc solid-phase peptide synthesis, purified using RP-HPLC, and the mass was analysed with MALDI-TOF mass spectrometry. The purified, reduced peptides were oxidised in ammonium bicarbonate with glutathione as a shuffling reagent, and a single major product was evident based on RP-HPLC analysis. This major isomer was purified and the mass confirmed. 

### 3.2. Structural Analysis

**Figure 2:**
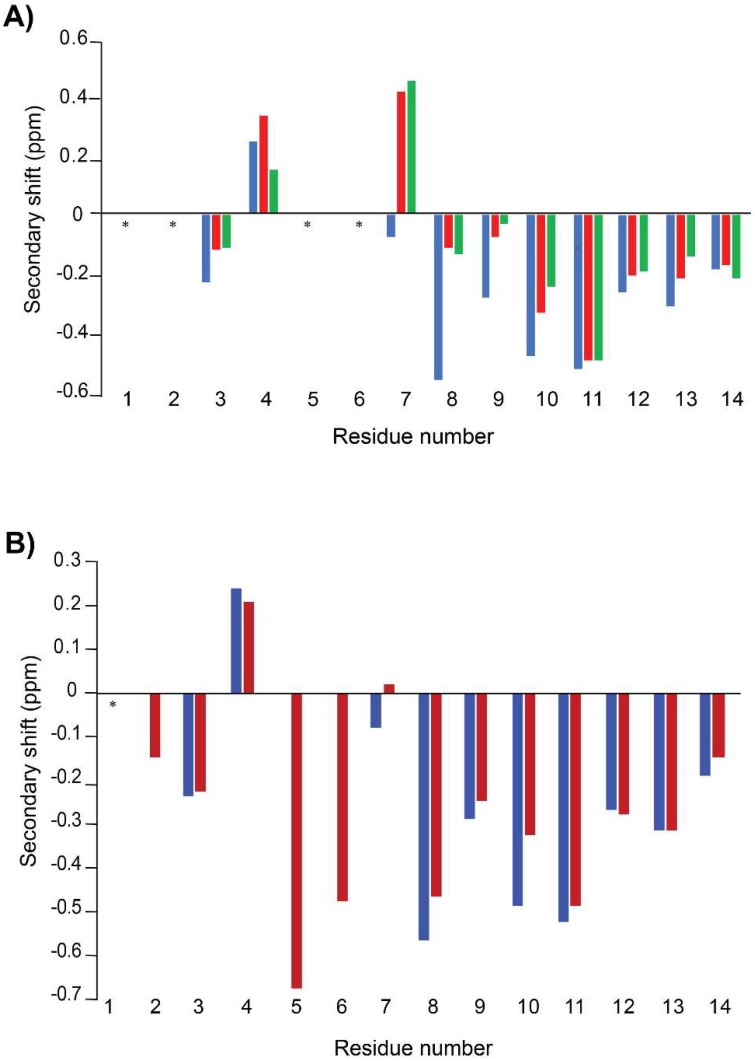
Secondary shift analysis. The secondary shifts were calculated by subtracting the random coil shifts [22] from the αH shifts. (**A**) MC12-linaclotide (blue), Linaclotide (red), and STh (6-19) (green); (**B**) Comparison of secondary shifts of MC12-linaclotide derived from spectra recorded in aqueous solution (blue) and 100 mM SDS (red). Residues that were not able to be assigned are marked with an asterisk.

The structures of linaclotide and MC12-linaclotide were analysed using NMR spectroscopy. NMR spectra were recorded in aqueous solution. Two-dimensional TOCSY and NOESY spectra allowed assignment of the majority of the resonances, and the secondary chemical shifts were determined by subtracting random coil chemical shifts from the αH chemical shifts [22]. Cys1, Cys2, Cys5, and Cys6 could not be assigned for either linaclotide or MC12-linaclotide. It is likely that these residues have very broad peaks, which prevented detection. A comparison of the secondary shifts for the assigned residues is shown in Figure 2A. The secondary shifts are similar between MC12-linaclotide and linaclotide, indicating that the peptides have the same overall fold. 

To confirm that the fold of the synthetic peptides was similar to related peptides, and therefore likely to have the native disulfide connectivity, we compared the secondary shifts to STh (6-19), which is a toxin that only differs from linaclotide by one residue; linaclotide contains a tyrosine at residue 4, whereas STh (6-19) contains a leucine. The NMR structure of STh (6-19) has previously been determined and the chemical shifts have been published [26]. STh (6-19) also has incomplete shifts for residues 1, 2, 5, and 6. All three peptides have similar secondary shifts consistent with them having the same disulfide connectivity (Cys1-Cys6, Cys2-Cys10, Cys5-Cys13) and overall fold. 

One-dimensional NMR spectra were recorded for MC12-linaclotide over the pH range 3.5 to 6 to determine if the missing resonances were evident. Peaks corresponding to the amide protons of residues 1, 2, 5, and 6 were not present in any of the spectra. In the one-dimensional spectrum recorded at pH 6, many of the amide protons were not evident, indicating that they had broadened beyond detection. 

**Figure 3:**
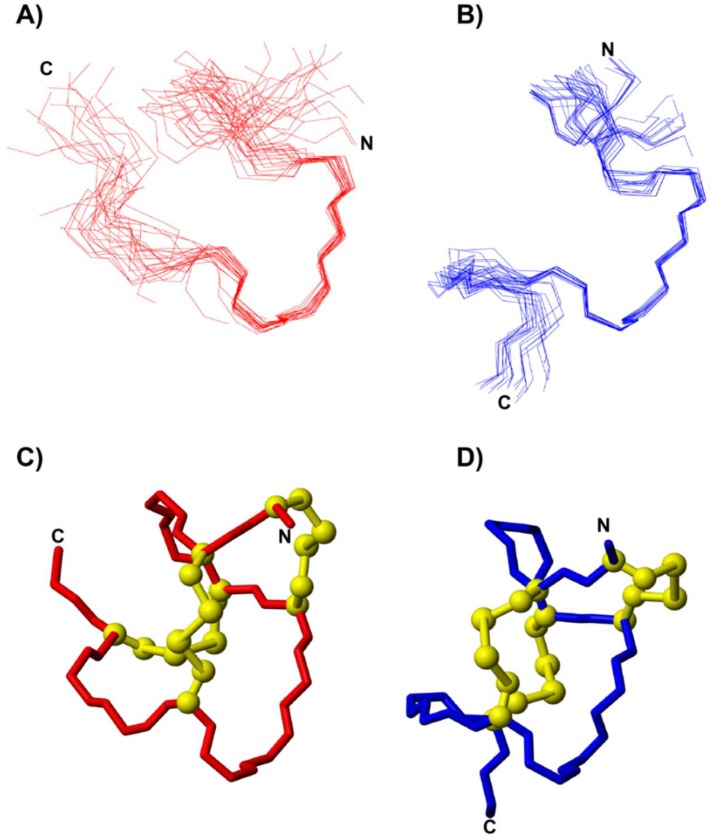
Three-dimensional structures of MC12-linaclotide. A superposition of the 20 lowest energy structures of MC12-linaclotide determined in (**A**) aqueous solution and (**B**) 100 mM SDS. (**C**) The lowest energy structure of MC12-linaclotide in (**C**) aqueous solution and (**D**) 100 mM SDS, with the disulfide bonds shown in ball-and-stick format. The figure was generated using MOLMOL [24].

The three-dimensional structure of MC12-lincalotide was calculated using CYANA [23,27], based on NOE and dihedral angle restraint data [28]. Although the N-terminal region is disordered, a 3_10_ helix from residues 7–10 was present based on analysis with MOLMOL. A superposition of the 20 lowest energy structures is given in Figure 3A, which highlights the disorder at the N- and C-termini, despite the high proportion of disulfide bonds in the peptide. 

To determine if a non-aqueous environment stabilizes the structure, spectra were recorded in the presence of 100 mM deuterated SDS. A comparison of the secondary shifts in aqueous solution and in SDS is given in Figure 2B. The shifts are similar between the two conditions, indicating that SDS does not significantly influence the overall fold. However, a larger number of NOEs were evident in the NOESY spectra recorded in the presence of SDS, and enabled a more well-defined structure to be calculated, as shown in Figure 3B.

### 3.3. TNBS Mouse Colitis Model

The effect of MC12-linaclotide in the TNBS-induced colitis mouse model was assessed. Mice were either left untreated (naïve), treated with TNBS alone, or were treated with peptides at a dose of 3 mg/kg five hours prior to the administration of TNBS. On day 3, mice were humanely euthanised using gas asphyxiation and examined for assessment of protection against colitis. 

**Figure 4:**
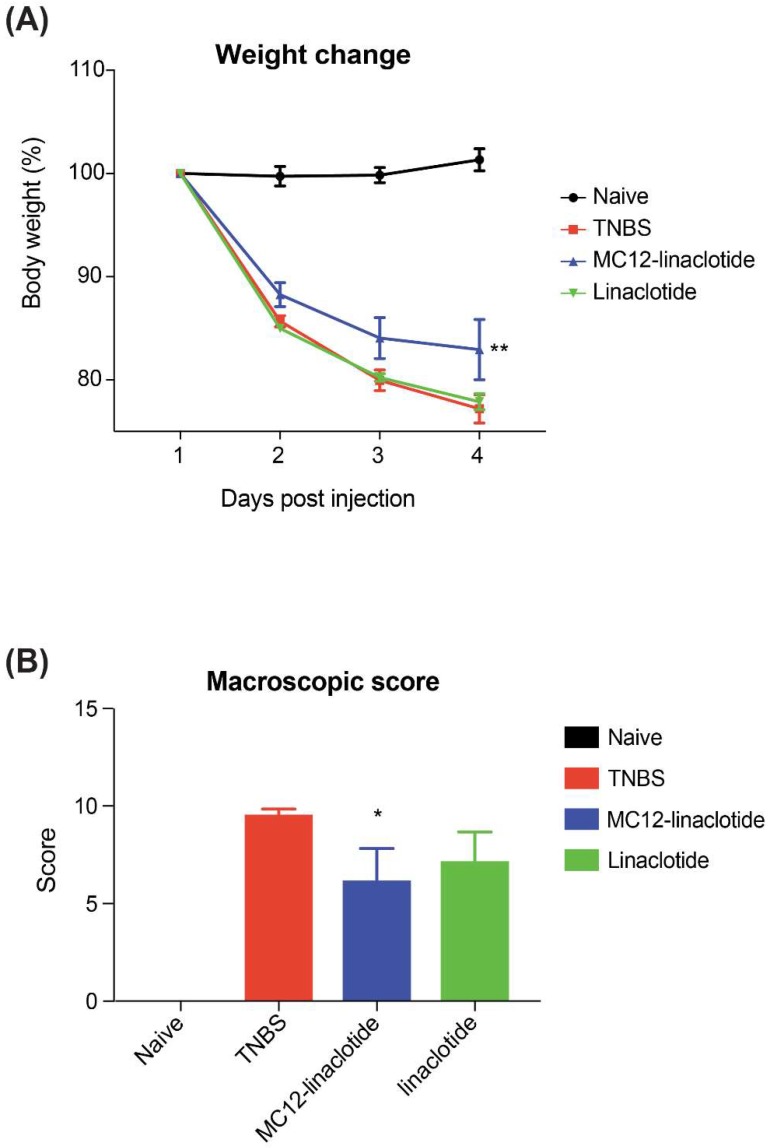
Protective effects of MC12-linaclotide against colitic weight loss and macroscopic pathology induced by intra-rectal administration of TNBS. Mice (5 per group) were untreated (naïve) or treated with TNBS following intra-peritoneal administration of peptides (3 mg/kg) or saline vehicle control (TNBS). (**A**) Percentage weight change (** *p* = 0.0018); (**B**) Macroscopic pathology score (* *p* = 0.0238). Data was analysed using GraphPad Prism. Statistical analyses of weights were performed using the two-way ANOVA, with multiple comparisons of the groups over different days. Macroscopic score was analysed using unpaired Mann-Whitney non-parametric tests. All values are expressed as mean ± SEM. Results were considered significant when *p* < 0.05.

The TNBS-treated mice lost weight and did not recover during the experiment, consistent with inflammation and colonic mucosa damage. By contrast, MC12-linaclotide-treated mice displayed statistically significant protective effects against weight loss (Figure 4A). MC12-linaclotide treatment resulted in statistically significantly improved macroscopic pathology scores compared to untreated mice that received TNBS (Figure 4B). No difference in colon lengths between groups was observed (results not shown). By contrast, linaclotide did not display protective effects, indicating that the grafting of the MC-12 sequence was responsible for the bioactivity.

### 3.4. Tissue p-IκB-α (Ser32) and p-NF-κB p65 (Ser536) Measurements

Analysis of colon tissue homogenates for phosphorylated transcription factor levels in mice [29] illustrates that linaclotide-treated mice (MC12-linaclotide + TNBS) have levels of phospho-NF-κB p65 (Ser536) and phospho-IκBα (Ser32) that are not statistically different to the TNBS-only treated mice. There appears to be a trend for lower levels of phosphorylated NFĸB in mice treated with MC12-linaclotide plus TNBS, which might suggest that the peptide is able to reduce the production of pro-inflammatory cytokines, but larger sample sizes may be required to reach statistical significance.

## 4. Discussion

Grafting bioactive sequences into peptide scaffolds is proving to be a useful approach for the design of novel drug leads [12,30,31]. Here, we show for the first time that the highly disulfide-rich peptide, linaclotide, can be used as a scaffold to confer anti-inflammatory activity in a TNBS mouse model of colitis.

The MC-12 tri-peptide has previously been shown to have protective effects in mouse models of colitis [32], and we have shown that grafting it into the SFTI-1 scaffold improves the potency and stability of the peptide [12]. Our current study confirms the importance of the MC-12 tri-peptide sequence (QAW), as linaclotide alone did not alleviate the symptoms of colitis, in contrast to the grafted MC12-linaclotide peptide which had a moderate but statistically significant influence on weight loss and macroscopic score. MC-12 is thought to interact with NF-κB and it is likely that the grafted peptides have the same mechanism of action, but this has yet to be explored. 

Analysis of the structures of the grafted MC12-linaclotide and linaclotide with NMR spectroscopy has been complicated due to the lack of spectral data in the N-terminal regions of both peptides. The missing peaks are most likely the result of structural flexibility in this region. Despite the incomplete assignments, the NMR analysis indicates that the synthetic forms of MC12-linaclotide and linaclotide used in the current study have similar overall folds. 

A comparison of the secondary shifts of linaclotide and MC12-linaclotide with a peptide closely related to linaclotide, a heat stable enterotoxin from the human strain of enterotoxigenic *E. coli* named STh (6-19)) [26], highlights the similarity with the synthetic peptides used in this study. Similar to the MC12-linaclotide peptide, STh (6-19) also has incomplete assignments for residues 1, 2, 5, and 6, suggesting that apparent flexibility in the N-terminal region is characteristic of this family of peptides. Overall, the NMR analysis indicates that our synthetic peptides have the same cysteine connectivity (Cys1-Cys6, Cys2-Cys10, Cys5-Cys13) as previously reported for linaclotide [33].

The three-dimensional structure of MC12-linaclotide was determined and as expected, was disordered at the N-terminus, but displayed a relatively well-defined region corresponding to the grafted MC-12 sequence, as shown in Figure 3A. The disorder at the N-terminus clearly results from the lack of assignments in the region, but this apparent flexibility is intriguing given the high percentage of cysteine residues in this peptide. 

A comparison of the secondary shifts of MC12-linaclotide in aqueous solution and 100 mM SDS (Figure 2B) highlights the similarity between the two conditions. However, in the presence of SDS, the α-protons of residues 2, 5, and 6 could be assigned, in contrast to the spectra recorded in aqueous solution. Peaks corresponding to these residues were not present in the aqueous solution data, and were broad in the SDS spectra. The structures determined in the presence of SDS are more well-defined than those in aqueous solution, indicating that SDS is having an impact on the dynamics of the peptide. 

**Figure 5:**
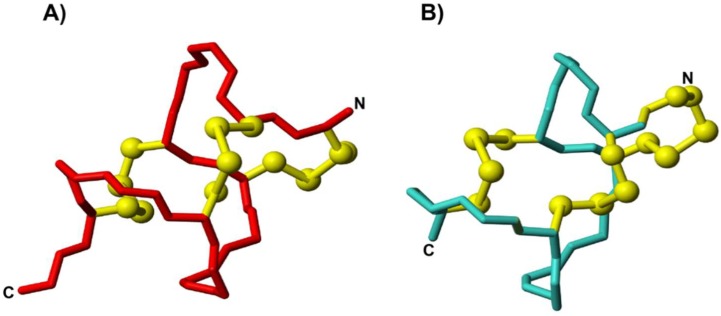
Three-dimensional structures of MC12-linaclotide and STp (5-17). (**A**) MC12-linaclotide determined in the presence of 100 mM SDS and (**B**) crystal structure of STp (5-17), PDB code: 1ETN. The figure was generated using MOLMOL [24].

A comparison of the three-dimensional structure of MC12-linaclotide in SDS with the crystal structure of a related peptide, STp (5-17), also highlights the similarity between the grafted peptide and peptides with similar sequences to linaclotide, as shown in Figure 5.

In summary, we have shown that linaclotide can serve as a scaffold to accommodate a small bioactive sequence and allow appropriate binding to a biological target. In particular, linaclotide is an interesting scaffold for the design of novel lead molecules for inflammatory bowel disease. Overall, this study provides further insight into grafting bioactive sequences into stable peptide scaffolds.
